# Gingival Periodontal Disease (PD) Level-Butyric Acid Affects the Systemic Blood and Brain Organ: Insights Into the Systemic Inflammation of Periodontal Disease

**DOI:** 10.3389/fimmu.2018.01158

**Published:** 2018-06-04

**Authors:** Marni E. Cueno, Kuniyasu Ochiai

**Affiliations:** Department of Microbiology, Nihon University School of Dentistry, Tokyo, Japan

**Keywords:** brain organ, butyric acid, inflammation, stress network, systemic blood

## Abstract

Butyric acid (BA) is produced by periodontopathic bacterial pathogens and contributes to periodontal disease (PD) induction. Moreover, PD has been associated with detrimental effects which subsequently may lead to systemic disease (SD) development affecting certain organs. Surprisingly, the potential systemic manifestations and organ-localized effects of BA have never been elucidated. Here, we simulated BA-based oral infection among young (20-week-old) rats and isolated blood cytosol to determine BA effects on stress network-related signals [total heme, hydrogen peroxide (H_2_O_2_), catalase (CAT), glutathione reductase (GR), free fatty acid (FFA), NADP/NADPH], inflammation-associated signals [caspases (CASP12 and CASP1), IL-1β, TNF-α, metallomatrix proteinase-9 (MMP-9), and toll-like receptor-2 (TLR2)], and neurological blood biomarkers [presenilin (PS1 and PS2) and amyloid precursor protein (APP)]. Similarly, we extracted the brain from both control and BA-treated rats, isolated the major regions (hippocampus, pineal gland, hypothalamus, cerebrum, and cerebellum), and, subsequently, measured stress network-related signals [oxidative stress: total heme, NADPH, H_2_O_2_, GR, and FFA; ER stress: GADD153, calcium, CASP1, and CASP3] and a brain neurodegenerative biomarker (Tau). In the blood, we found that BA was no longer detectable. Nevertheless, oxidative stress and inflammation were induced. Interestingly, amounts of representative inflammatory signals (CASP12, CASP1, IL-1β, and TNF-α) decreased while MMP-9 levels increased which we believe would suggest that inflammation was MMP-9-modulated and would serve as an alternative inflammatory mechanism. Similarly, TLR2 activity was increased which would insinuate that neurological blood biomarkers (APP, PS1, and PS2) were likewise affected. In the brain, BA was not detected, however, we found that both oxidative and ER stresses were likewise altered in all brain regions. Interestingly, tau protein amounts were significantly affected in the cerebellar and hippocampal regions which coincidentally are the major brain regions affected in several neurological disorders. Taken together, we propose that gingival BA can potentially cause systemic inflammation ascribable to prolonged systemic manifestations in the blood and localized detrimental effects within the brain organ.

## Introduction

Periodontal diseases (PD) are often associated with periodontopathic bacteria found in the host oral cavity (1). Moreover, PD was previously suggested to cause detrimental systemic manifestations leading to systemic disease (SD) development that would affect certain organs (2). Oxidative stress has been linked to endoplasmic reticulum (ER) stress (3) and both stresses have been correlated to SD (2, 4). This would emphasize the significance of PD-induced oxidative and ER stresses to SD development.

High concentrations of secondary metabolites produced by periodontopathic bacteria are often linked to PD development and, likewise, are detected in the oral cavity of patients with severe PD (5). One example of a periodontopathic bacterial secondary metabolite is butyric acid (BA) which is a short chain fatty acid (SCFA) produced either through the butyrate kinase or butyryl-CoA: acetate CoA-transferase pathways (6). Previously, BA has been reported to have both beneficial (energy source for colorectal cells, helps the intestine maintain colonic health, and positively influence immune responses) and detrimental (PD development) effects depending on what tissue BA accumulates in Ref. (7, 8). In an earlier work, we showed that BA has prolonged retention within the gingival tissue which in turn gradually enters the blood stream inducing oxidative and ER stresses in the systemic rat blood (9–11). This would mean that BA-related PD induction may likewise serve as a risk factor and have detrimental systemic manifestations; however, this was never elucidated. Additionally, it was previously suggested that periodontopathic bacteria can reach the brain *via* systemic circulation (12) which would similarly insinuate that bacterial products (such as BA) could also reach the brain *via* the same mechanism. Similarly, this was never elucidated. A better understanding of the possible systemic manifestations and organ-localized effects of BA would highlight the potential role of periodontopathic bacterial products in SD development and, likewise, could lead to therapeutic strategies that aim to hamper bacteria-associated SD development.

## Materials and Methods

### Animal Handling and Sample Collection

Throughout this study, handling and treatment of male Wistar rats were made according to previously published works (9, 10). Briefly, 10-week-old male Wistar rats (Japan SLC, Shizuoka, Japan) were housed individually in stainless steel cages with wire-mesh bottoms and placed in a room under controlled conditions [temperature (23–25°C), relative humidity (40–60%), and lighting (12 h)]. Rats freely accessed water and were fed a semi-purified diet following the AIN93G formulation. Initially, rats were acclimatized for 7 days prior to the actual study. Handling of rats was in accordance with the animal study guidelines of the Kyoto Institute of Nutrition and Pathology. Acclimatized rats (*n* = 6) were implanted with jugular canulae under sodium pentobarbital anesthesia (40 mg kg^−1^ body weight) and 10 µL of ^13^C *n*-butyrate solution (1 M) was injected in several batches into the gingival mucosa until obtaining the same periodontal disease level-butyric acid (PDL-BA) levels found among patients with periodontitis (5 mM) (13). We opted to use sodium butyrate in order to avoid other physiological changes associated by using BA. BA concentration in the blood was verified through LC–MS as previously described (9–11). Control jugular blood was collected prior to BA injection (0 h). BA-treated jugular blood was collected 6 and 12 h after BA injection for use in further downstream analyses. Blood collection times are 2× and 4× longer compared to the longest time (3 h) in our earlier works (9–11). Similarly, control and BA-treated rat brains were collected and subdivided into the hippocampus, pineal gland, hypothalamus, cerebrum, and cerebellum for further downstream analyses. Moreover, LC–MS was used to confirm BA presence in the different brain regions.

### Blood and Brain Sample Processing

For analysis using the rat blood, all samples were utilized and the main focus was on the blood cytosol which refers to the extracted cytosolic components of all cells in the blood. Both isolation and purity were established as previously described (10). Cytosol/Particulate Rapid Separation Kit (BioVision Inc., CA, USA) was used to isolate the blood cytosolic components. For analysis using the different brain regions (hippocampus, pineal gland, hypothalamus, cerebrum, and cerebellum), only control and 12 h post-treatment with PDL-BA were used for analyses. Each brain part was further cut into 1–2 mm length tissue samples and placed in a sterile microtube containing N-PER^TM^ Neuronal Protein Extraction Reagent (Thermo Scientific, CA, USA) prior to homogenization using a handheld motorized homogenizer. For both processed blood and brain samples, Pierce^®^ Detergent Removal Spin Columns (Thermo Scientific) and Pierce^®^ Microplate BCA Protein Assay Kit-Reducing Agent Compatible Kit (Thermo Scientific) were used to purify and standardize blood cytosol samples prior to downstream analyses. All kits were used according to manufacturer’s recommendation.

### Quantification of Selected Biochemical Components in the Blood

Biochemical components quantified in the blood cytosol are classified as: stress-linked components [total heme, hydrogen peroxide (H_2_O_2_), catalase (CAT), NADP and NADPH, free fatty acid (FFA), and glutathione reductase (GR)], and inflammation-linked components [caspase-12 (CASP12), caspase-1 (CASP1), IL-1β, and matrix metalloproteinase-9 (MMP-9)].

For determining the concentration of stress-linked components, QuantiChrom™ Heme Assay Kit (BioAssay Systems, CA, USA), Red Hydrogen Peroxide Assay Kit (Enzo Life Sciences, PA, USA), EnzyChrom™ Catalase Assay Kit (BioAssay Systems), NADP^+^/NADPH Assay Kit (BioAssay Systems), EnzyChrom™ Free Fatty Acid Assay Kit (BioAssay Systems), and Glutathione Reductase Activity Colorimetric Assay Kit (BioVision) was used to determine total heme levels (free heme and heme-proteins), H_2_O_2_, CAT, NADPH/NADP, FFA, and GR amounts, respectively. All kits were used according to manufacturer’s recommendation.

For establishing the amounts of inflammation-linked components, Caspase-12 Fluorometric Assay Kit (BioVision Inc.), Caspase-1/ICE Colorimetric Assay Kit (BioVision Inc.), RayBio^®^ Rat IL-1 beta ELISA Kit (RayBio, Inc., GA, USA), and Quantikine^®^ ELISA Rat Total MMP-9 Immunoassay (R&D Systems, Inc., MN, USA) was utilized to quantify CASP12, CASP1, IL-1β, and MMP-9 amounts, respectively. Similarly, all kits were used according to manufacturer’s recommendation. Additionally, toll-like receptor-2 (TLR2) amounts were determined using ELISA. Briefly, antigen and sodium bicarbonate-sodium carbonate buffer (Polysciences, Inc., Taipei, Taiwan) solution was used to coat the desired antigen (at 1 µg mL^−1^ concentration) onto the polystyrene plates and overnight blocking was performed using a commercially available PBS with 1% BSA blocking buffer (GeneTex). Recombinant TLR2 protein (Abnova, Taipei, Taiwan) was used to establish a protein standard, whereas, primary TLR2 polyclonal antibody (Bioss Inc., MA, USA) and rabbit secondary antibody (GE Healthcare, Little Chalfont, United Kingdom) were utilized to detect blood cytosolic TLR2 levels. SIGMA*FAST*™ OPD tablets (Sigma-Aldrich Co., MO, USA) was utilized for peroxidase detection. Washing in-between steps was done using the PBS/Tween^®^ Solution (AppliChem GmbH, Darmstadt, Germany) and hydrochloric acid (1.0 M) was used as a stop solution. ELISA measurements were done in Abs 450 nm.

### Detection of Representative Blood Biomarkers

Amyloid precursor protein (APP) and both presenilin-1 (PS1) and presenilin-2 (PS2) were selected as blood biomarkers, since all three components are also known to affect the brain (14–16). Western blotting was performed using anti-APP (GeneTex, Inc., CA, USA), anti-PS1 (Novus Biologicals, CO, USA), and anti-PS2 (GeneTex) to detect and estimate APP, PS1, and PS2 amounts in the blood cytosol, respectively. Anti-GAPDH (GeneTex) was used as control. Briefly, protein samples were initially separated by SDS-PAGE and, subsequently, transferred to Hybond-C nitrocellulose membrane (Amersham Biosciences Corp., NJ, USA). Membranes were blocked with Difco™ Skim Milk (BD Company, Erembodegem, Belgium), probed with antibodies, and immunoreactive proteins were visualized using SuperSignal^®^ West Pico Chemiluminescent Substrate (Thermo Scientific).

### Measuring of Selected Biochemical Components in the Varying Brain Regions

Biochemical components studied are classified as: oxidative stress-related (total heme, NADPH, H_2_O_2_, GR, and FFA amounts), ER stress-linked (GADD153, calcium), and cell death-associated (CASP1, CASP3). Oxidative stress-related components were measured as earlier discussed. For ER stress-linked components, GADD153 levels were quantified through ELISA. Briefly, coating and blocking of ELISa plates were as earlier described. Recombinant GADD153 protein (GeneTex Inc.) was used to establish a protein standard, whereas, HRP-conjugated GADD153/CHOP antibody (Novus Biologicals) was utilized to detect GADD153 levels. Peroxidase detection and washing in-between steps was as earlier described. Hydrochloric acid (1.0 M) was used as a stop solution. ELISA measurements were done in Abs 450 nm. Calcium Colorimetric Assay Kit (BioVision) was used to measure calcium amounts in the brain. For cell death-associated components, CASP1 (representing inflammation) and CASP3 (representing apoptosis) were quantified using Caspase-1/ICE Colorimetric Assay Kit and Caspase-3/CPP32 Colorimetric Assay Kit, respectively. All kits were used according to manufacturer’s recommendation.

### Representative Brain Biomarker That Is Potentially Affected

Tau protein plays a role in neurodegenerative disorders (17) making it an ideal brain biomarker to establish any potential detrimental effects of PDL-BA on the brain. A similar ELISA protocol was performed as earlier described. Tau protein (Aviva System Biology, CA, USA) was used to develop a protein standard, while, primary Tau polyclonal antibody (Bioss Inc., MA, USA) and rabbit secondary antibody were utilized to quantify Tau levels in the particular brain region in both control and BA-treated rats.

### Network Analysis of the Blood and Brain Components

Network analysis is an important aspect of systems biology and several computer-based modeling environments have been developed to assist in understanding the network of various biochemical components involved (18). We used Cytoscape to understand the network of biochemical components studied (19). For this study, we focused on the following topological centralities: (1) stress centrality to establish the involvement of each component with regards to the whole network; (2) betweenness centrality to establish how crucial a component is to the whole network; (3) closeness centrality to determine the relevance of a component studied; (4) radiality centrality to further determine whether a component is relevant or irrelevant; (5) eccentricity centrality to determine how readily accessible a component is with regards to the other components; and (6) edge betweenness centrality to elucidate the importance between two components (20). Briefly, we first established the threshold for each centrality and values above the threshold were considered significant edges and nodes.

### Statistical Analyses

Statistical analyses were performed first using the Andersen–Darling normality test to check whether the values obtained were normalized in both the blood and brain samples. If *p* > 0.05, the values obtained were considered acceptable and statistical significance of differences was further elucidated using Student’s *t*-test, wherein, a significance level of 95% (*p* < 0.05) was considered statistically significant.

### Statement on Reproducibility

All assays performed were conducted in duplicate in order to establish replicability of results (results reproducibility) and, more importantly, results obtained were confirmed through consilience (each data supporting the other) which is consistent with inferential reproducibility (21, 22).

## Results

### Gingival PDL-BA Induces Oxidative Stress in the Blood Cytosol

Initially, LC–MS was performed to confirm the presence and concentration of BA in the blood. We found that BA was no longer detected at 6 and 12 h post-BA injection (data not shown). Nevertheless, after sample processing, we performed subsequent assays in order to elucidate the possible prolonged effects of BA.

**Figure 1:**
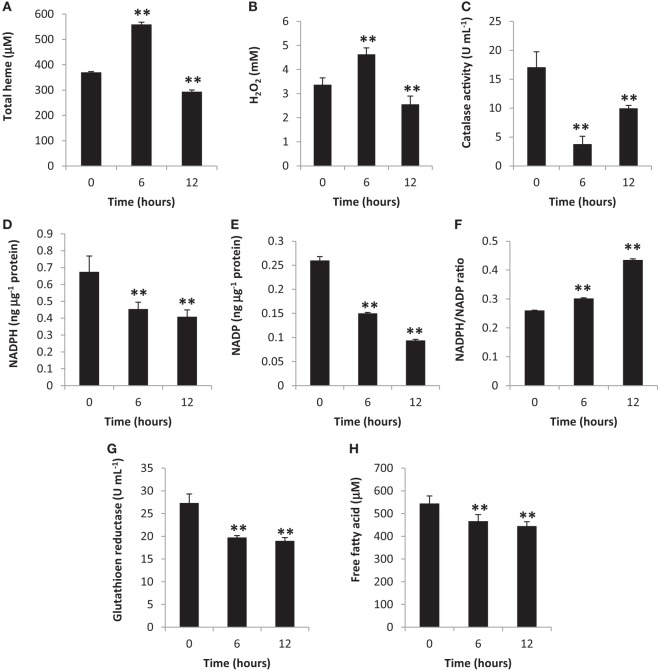
Gingival periodontal disease level-butyric acid (BA) induces blood cytosolic oxidative stress. Quantification of blood cytosolic **(A)** total heme, **(B)** H_2_O_2_, **(C)** catalase, **(D)** NADPH, **(E)** NADP, **(F)** NADPH/NADP ratio, **(G)** free fatty acid, and **(H)** glutathione reductase amounts are indicated. Results shown are mean ± SE utilizing independent blood samples (collected at 0, 6, and 12 h post-treatment with BA) of 10-week-old Wistar male rats (*n* = 6). Statistical analyses were performed using Anderson–Darling normality test and, if passed (*p* > 0.05), Student’s *t*-test (**p < 0.01).

Heme is one of the most important biomolecules, since it is involved in an array of biological reactions with free heme and heme-proteins having been related to oxidative stress induction (23, 24). Additionally, other components involved in oxidative stress is a pro-oxidant (such as H_2_O_2_) (25) and an anti-oxidant (such as CAT) (26). To determine oxidative stress induction, we measured total heme, H_2_O_2_, and CAT amounts in the blood cytosol. We observed that all three components were altered at 6 and 12 h post-BA injection (Figures 1A–C, respectively) which would suggest that oxidative stress was induced.

To further confirm oxidative stress induction in the blood cytosol, we likewise measured both NADPH and NADP amounts and, subsequently, computed the NADPH/NADP ratio which is another indicator of oxidative stress induction (27). We observed that blood cytosolic NADPH (Figure 1D) and NADP (Figure 1E) amounts were decreased suggesting that these components were consumed possibly for NADPH-related biochemical processes (which includes pro-oxidation). In contrast, the NADPH/NADP ratio was increased (Figure 1F) among BA-treated rats as compared to the control insinuating that oxidative stress was induced consistent with our earlier results (Figures 1A–C). NADP is utilized to synthesize NADPH, whereas, NADPH contributes to pro-oxidation, anti-oxidation, and biomolecule synthesis (28, 29). Considering oxidative stress was induced at both 6 and 12 h post-BA injection, we likewise suspected that BA-linked decrease in NADPH and NADP amounts similarly affected NADPH-related anti-oxidants (such as GR) and biomolecules (such as FFA) synthesis.

To determine the blood cytosolic GR and FFA levels, both GR and FFA were quantified. As shown in Figure 1G (GR) and Figure 1H (FFA), both blood cytosolic components were decreased among BA-treated rats consistent with decreased NADPH amounts (Figure 1D). We postulate that at both 6 and 12 h post-BA injection, NADPH was mainly utilized for pro-oxidant synthesis (H_2_O_2_) consequentially resulting to decreased NADPH amounts available for anti-oxidant (GR) and biomolecule (FFA) synthesis.

It is worth mentioning that in our earlier works (9, 10) PDL-BA was still detectable in the blood until 3 h after BA injection in the gingival tissue, whereas, in this study, PDL-BA was no longer detectable in 6 and 12 h after BA injection. In this regard, we attributed the possible contradicting oxidative stress-related data which we observed in the blood between our earlier works and this study to BA presence.

### Blood Cytosolic Inflammatory Caspase and Cytokine Activities are Decreased

Oxidative stress induction is associated with inflammation (30). In a previous work, we showed that gingival PDL-BA induced oxidative stress and, subsequently, ER stress 3 h post-BA injection which in-turn activated cell death signaling (apoptosis and inflammation) (11). In relation to this and in-line with our earlier results, we likewise investigated whether inflammatory signals were activated in the blood cytosol of BA-treated rats.

**Figure 2:**
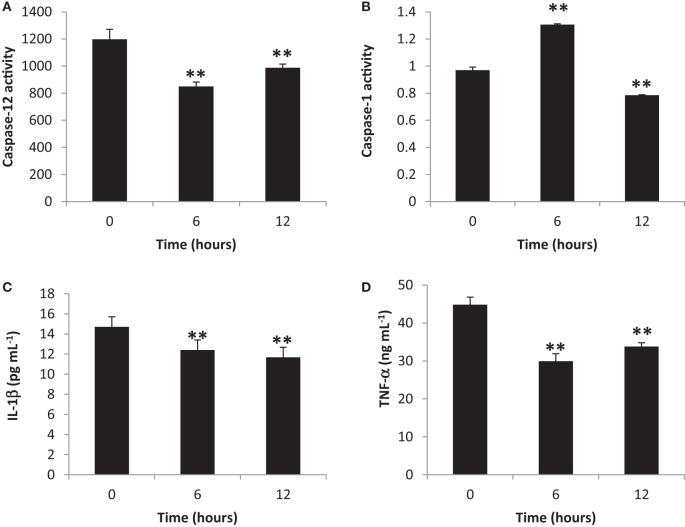
Inflammatory caspase and cytokine activities in the blood cytosol are decreased ascribable to gingival periodontal disease level-butyric acid (BA). Quantification of blood cytosolic **(A)** caspase-12 activity, **(B)** caspase-1 activity, **(C)** IL-1β amounts, and **(D)** TNF-α amounts are indicated. Results shown are mean ± SE utilizing independent blood samples (collected at 0, 6, and 12 h post-treatment with BA) of 10-week-old Wistar male rats (*n* = 6). Statistical analyses were performed using Anderson–Darling normality test and, if passed (*p* > 0.05), Student’s *t*-test (**p < 0.01).

To elucidate the activation of blood cytosolic caspases by gingival PDL-BA, we measured blood cytosolic CASP12 and CASP1 amounts. We found that CASP12 activity decreased at 6 and 12 h post-BA injection (Figure 2A), while, CASP1 activity increased and decreased at 6 and 12 h post-BA injection, respectively (Figure 2B). CASP12 is a known regulator of CASP1 (31). In this regard, we believe that at 6 h post-BA injection, decrease in CASP12 activity allowed for an increase in CASP1 activity. Moreover, both CASP12 and CASP1 were decreased at 12 h post-BA injection which we think is attributable to maintenance of organismal homeostasis, whereby, both CASP12 and CASP1 activities are decreased in order to improve disease outcomes and alleviate disease pathologies (32).

To establish the effects of gingival PDL-BA on representative blood cytosolic pro-inflammatory cytokines, we quantified blood cytoslic IL-1β and TNF-α activities. We observed decreased IL-1β (Figure 2C) and TNF-α (Figure 2D) activities among BA-treated rats. This would mean that pro-inflammatory cytokines were not activated regardless of PDL-BA-linked oxidative stress induction. Considering oxidative stress was induced (Figures 1A,F) and pro-inflammatory cytokine activities were decreased (Figures 2C,D), we hypothesize that an alternative mechanism or pathway not involving cytokines could have signaled inflammation.

It is worth mentioning that at 6 h post-BA injection, IL-1β activity was decreased regardless of an increase in CASP1 activity (Figure 2B). CASP1 is known to activate IL-1β which is involved in inflammation (33) and, similarly, sterol regulatory element binding proteins which is involved in cholesterol and fatty acid biogenesis (32). We suspect that the increased CASP1 activity at 6 h post-BA injection is not related to inflammation [consistent with decreased IL-1β activity (Figure 2C)] but instead is putatively associated with cholesterol and fatty acid biogenesis. This would further emphasize that caspase activation is not essential for initiating inflammation as indicated in an earlier publication (34).

### MMP-9-Modulated Inflammation Affects Neurological Blood Biomarkers

**Figure 3:**
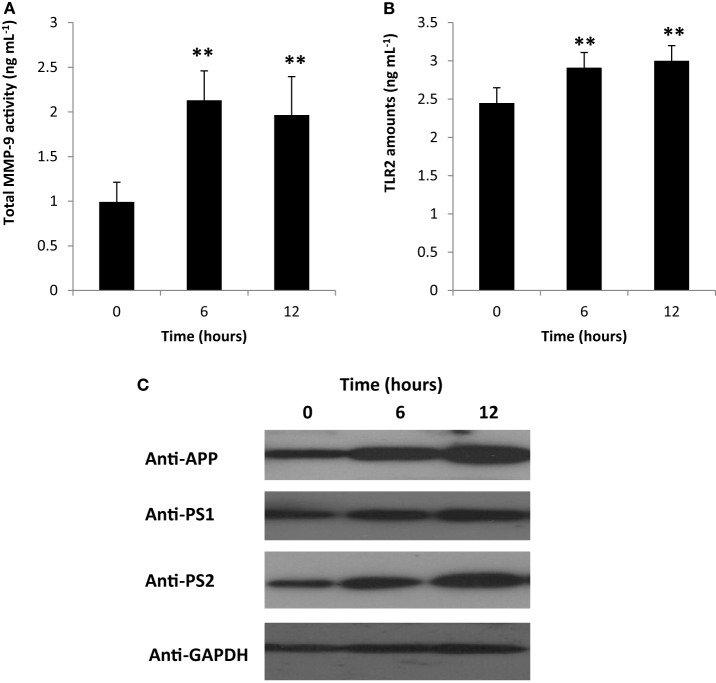
Gingival periodontal diseases level-butyric acid (BA) affected matrix metalloproteinases (MMP)-9-modulated inflammation affected representative neurological blood biomarkers. Quantification of blood cytosolic **(A)** MMP-9 activity and **(B)** toll-like receptor-2 (TLR2) amounts are indicated. Results shown are mean ± SE utilizing independent samples (collected at 0, 6, and 12 h post-treatment with BA) of 10-week-old Wistar male rats (*n* = 6). Statistical analyses were performed using Anderson–Darling normality test and, if passed (*p* > 0.05), Student’s *t*-test (**p < 0.01). **(C)** Western blotting data of representative blood biomarkers. Antibodies used to detect amyloid precursor protein (APP), presenilin-1 (PS1), preseinilin-2 (PS2), and glyceraldehydes-3-phosphate (GAPDH) are indicated.

Matrix metalloproteinases (MMP) are enzymes responsible for the degradation of extracellular matrix and play a role in various biological activities and pathological processes (which include inflammation) (35–38). Moreover, MMPs have dual function and are involved in both inflammatory modulation and inactivation (39). Among the known MMPs, MMP-9 activity has been linked to inflammation-related pathologies (37). To elucidate the possible role of PDL-BA in modulating inflammation, we measured blood cytosolic MMP-9 activity. We observed that blood cytosolic MMP-9 activity was increased 6 and 12 h post-BA injection (Figure 3A). This would suggest that MMP-9 plays an important role in PDL-BA-associated modulation of inflammation. Coincidentally, MMP-9 activity is regulated by both IL-1β and TNF-α (40). In this regard and considering both cytokine activities were decreased (Figures 2C,D) while MMP-9 activity was increased (Figure 3A), we suspect that MMP-9-modulated inflammation is the alternative mechanism involved in signaling inflammation during PDL-BA-associated oxidative stress induction. Additionally, MMP-9 activation has been linked to TLR2 activity (41) which would imply that blood cytosolic TLR2 amounts were likewise affected.

To determine the amount of TLR2 in the blood cytosol, we quantified blood cytosolic TLR2 amounts using ELISA. We found that TLR2 levels were increased in both 6 and 12 h post-BA injection (Figure 3B) consistent with MMP-9 amounts (Figure 3A). This would insinuate that during an MMP-9-modulated inflammation, TLR2 activity is likewise increased. TLR2 activation is triggered by damaged tissues or cells and is involved in the induction of neuroinflammatory responses (42, 43). We suspect that increased TLR2 activity may likewise affect neurological components that can be found in the blood.

To elucidate whether neurological blood biomarkers were affected by gingival PDL-BA *via* TLR2 activation, Western blotting of APP, PS1, and PS2 proteins were performed. As seen in Figure 3C, approximate protein concentration of all three representative blood biomarkers (APP, PS1, PS2) were increased at 6 and 12 h post-BA injection consistent with TLR2 activation.

### Gingival PDL-BA Induces Oxidative Stress within the Different Regions of the Brain Organ

Similar to blood analysis, LC–MS was first performed to confirm the presence and concentration of BA in the different parts of the brain. Consistently, we found that at 6 and 12 h post-BA injection no BA was detected (data not shown). Considering the results obtained from the blood cytosol, we likewise performed additional assays using the different brain regions.

**Figure 4:**
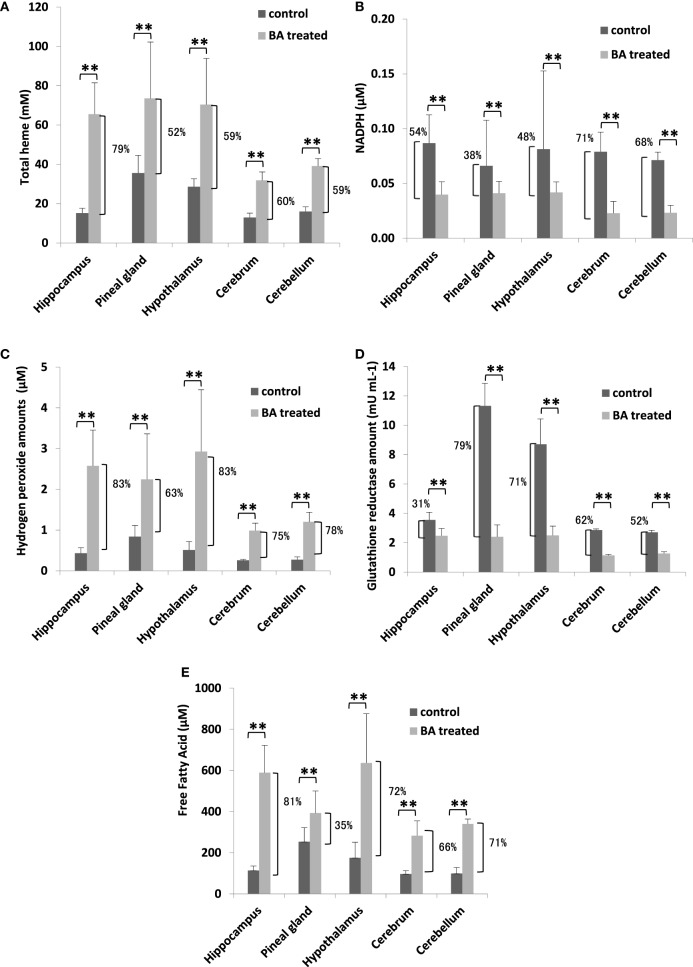
Gingival periodontal disease level-butyric acid (BA) induces oxidative stress in different regions of the brain organ. Quantification of brain **(A)** total heme, **(B)** NADPH, **(C)** hydrogen peroxide, **(D)** glutathione reductase, and **(E)** free fatty acid amounts are indicated. Results shown are mean ± SE utilizing independent brain samples (hippocampus, pineal gland, hypothalamus, cerebrum, and cerebellum) of 10-week-old Wistar male rats (*n* = 6). Control and BA-treated (12 h post-treatment) rats were used. Statistical analyses were performed using Anderson–Darling normality test and, if passed (*p* > 0.05), Student’s *t*-test (**p < 0.01). Percent difference between control and BA-treated rats are labeled.

To establish the possible neurological effects of gingival PDL-BA, we measured components related to oxidative stress induction in the different regions of the brain. We found that total heme amounts were increased (Figure 4A), whereas, NADPH levels were decreased (Figure 4B). These results were consistent with our earlier results in blood (Figures 1A,D). Additionally and as we have earlier mentioned, NADPH contributes to pro-oxidation (H_2_O_2_), anti-oxidation (GR), and biomolecule synthesis (FFA) (28, 29). In this regard, we attributed the decrease in brain NADPH levels to increased brain H_2_O_2_ (Figure 4C), GR (Figure 4D), FFA (Figure 4E) amounts in the different regions of the brain. Concurrent increase in H_2_O_2_ and GR levels are consistent with BA-related oxidative stress induction (10). Surprisingly, brain FFA levels were increased among BA-treated rats (Figure 4E). FFA can serve as either a physiological fuel or, simultaneous with oxidative stress induction, contribute to disease pathology (44). We suspect that increased brain FFA amounts serve a pathological function (consistent with oxidative stress induction) in the different regions of the brain.

### Brain ER Stress Stimulation and Cell Death Activation are Ascribable to Gingival PDL-BA

Endoplasmic reticulum stress which occurs when organelle function is impaired by either physiological or pathological conditions (45). PD is associated with both oxidative stress and SD (2, 4) while oxidative stress is correlated to ER stress (3). Considering brain oxidative stress was induced attributable to gingival PDL-BA, we likewise postulate that ER stress stimulation and cell death activation occurred.

**Figure 5:**
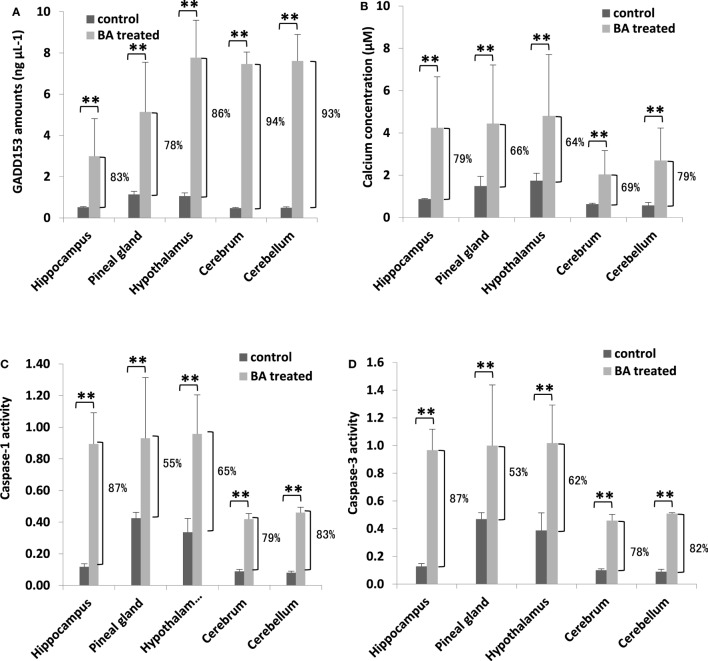
Endoplasmic reticulum stress stimulation and cell death activation in the different brain regions are ascribable to gingival periodontal disease level-butyric acid (BA). Quantification of brain **(A)** GADD153 amounts, **(B)** calcium concentration, **(C)** caspase-1 activity, and **(D)** caspase-3 activity are indicated. Results shown are mean ± SE utilizing independent brain samples (hippocampus, pineal gland, hypothalamus, cerebrum, and cerebellum) of 10-week-old Wistar male rats (*n* = 6). Control and BA-treated (12 h post-treatment) rats were used. Statistical analyses were performed using Anderson–Darling normality test and, if passed (*p* > 0.05), Student’s *t*-test (**p < 0.01). Percent difference between control and BA-treated rats are labeled.

To determine whether brain ER stress was stimulated, we measured brain GADD153 and calcium amounts. Similarly, cell death activation (inflammation: CASP1; apoptosis: CASP1) were likewise quantified. We observed that both brain GADD153 (Figure 5A) and calcium (Figure 5B) amounts were increased among BA-treated rats (Figure 5A). In addition, CASP1 (Figure 5C) and CASP3 (Figure 5D) activities among BA-treated rats were likewise increased. GADD153 is used as an ER stress marker (46) while ER stress has been linked to calcium signaling (47). Moreover, calcium signaling has been associated with cell death activation, particularly apoptosis and inflammation (48, 49). These results would imply that brain ER stress was stimulated possibly ascribable to gingival PDL-BA. ER stress causes abnormal conformations of proteins to accumulate which in-turn threaten cellular survival leading to irreparable damage to cellular function (45). Several neurodegenerative diseases are attributable to pathological forms of proteins accumulating and depositing in the brain (50). In this regard and considering brain ER stress was stimulated, we suspect that the gingival PDL-BA has an unfavorable effect in the brain.

### Gingival PDL-BA Affects Brain Tau Protein Amounts

**Figure 6:**
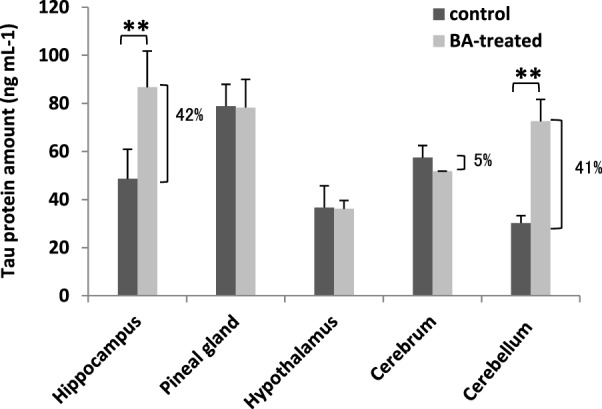
Brain tau protein amounts are affected by gingival periodontal disease level-butyric acid (BA). Measurement of brain tau protein amounts is indicated. Results shown are mean ± SE utilizing independent brain samples (hippocampus, pineal gland, hypothalamus, cerebrum, and cerebellum) of 10-week-old Wistar male rats (*n* = 6). Control and BA-treated (12 h post-treatment) rats were used. Statistical analyses were performed using Anderson–Darling normality test and, if passed (*p* > 0.05), Student’s *t*-test (**p < 0.01). Percent difference between control and BA-treated rats are labeled.

Under normal conditions, tau protein is expressed abundantly within neurons and is responsible for stabilizing the neuronal microtubule network in the brain (51). In contrast, under pathological conditions, tau proteins have decreased solubility allowing it to aggregate and resulting to varying disease tauopathies (52). This makes tau proteins an ideal biomarker for detecting certain brain disorders, including neuronal disorders (17). To elucidate the effects of gingival PDL-BA on the brain, we measured tau protein amounts in the varying brain regions. As shown in Figure 6 and among the different brain regions isolated, increased tau protein amounts were observed in the brain hippocampus and cerebellum which would insinuate that these regions are most affected by gingival PDL-BA. In contrast, no significant changes were observable in the pineal gland, hypothalamus, and cerebrum regardless of brain ER stress stimulation. Considering distinct cell types may have unique, deficient, or developmentally regulated responses to modulate ER stress stimulation (53), we believe that ER stress stimulated in the pineal gland, hypothalamus, and cerebrum have a more developed ER stress response mechanism compared to the hippocampus and cerebellum. Admittedly, additional experimentation (such as histological studies) is needed to confirm these results.

### Holistic and Centrality Analysis of All Components Affected by Gingival PDL-BA

In order to elucidate the holistic effect of gingival PDL-BA, each network element has to be studied and, similarly, the interrelationship between these elements has to be established (20, 54). Centrality analysis is one method that allows for the ranking of network elements in order to identify elements of interest found within a network (20). In this regard, we designed the blood and brain network based on the various representative biochemical components studied.

**Figure 7:**
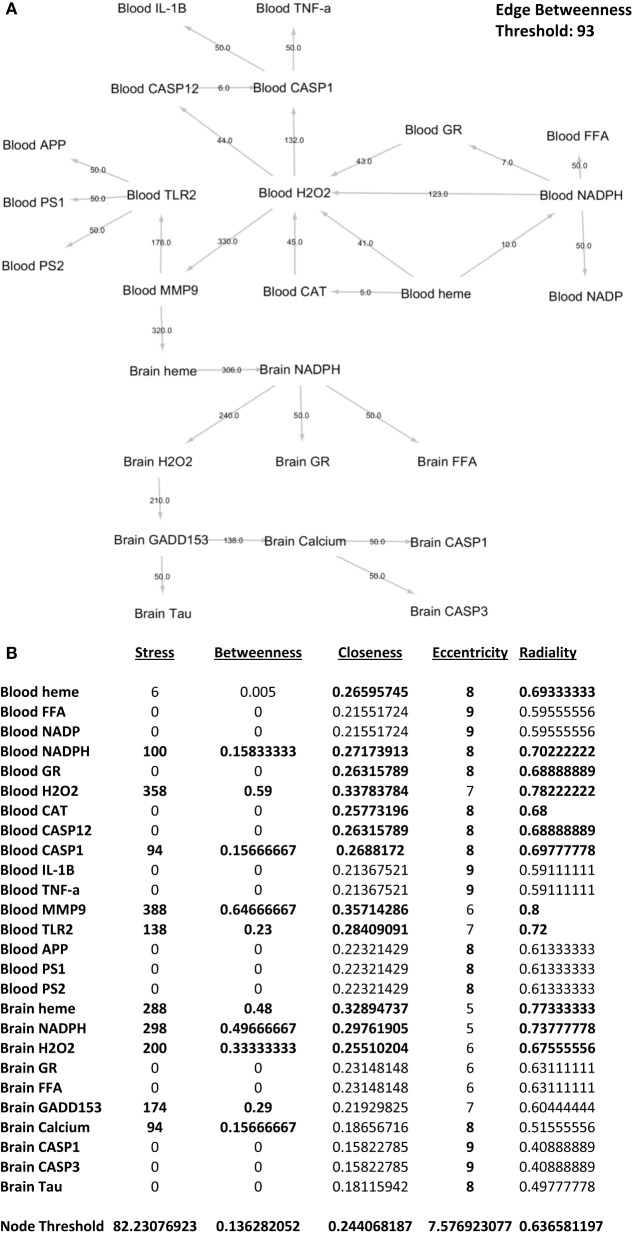
Network analyses of representative biochemical components affected by gingival periodontal disease level-butyric acid. **(A)** Network design of both the blood and brain biochemical components. Edge betweenness centrality data analyses are indicated over the arrows. Edge betweenness threshold is shown. **(B)** Centrality analyses of the blood and brain biochemical components. Values for stress, betweenness closeness, eccentricity, and radiality centralities of each component are indicated. Node threshold for each centrality measurement is shown.

To identify which interactions between biochemical components are significant with regards to the overall network, we performed edge betweenness centrality analysis. As seen in Figure 7A, the following interactions were considered significant relative to the overall network: blood NADPH–blood H_2_O_2_, blood H_2_O_2_–blood CASP1, blood H_2_O_2_–blood MMP-9, blood MMP-9–blood TLR2, blood MMP-9–brain heme, brain heme–brain NADPH, brain NADPH–brain H_2_O_2_, brain H_2_O_2_–brain GADD153, and brain GADD153–brain calcium. This would imply that these interactions were affected by gingival PDL-BA. In addition, we identified the series of networks affected by gingival PDL-BA that connected blood NADPH to brain calcium. More importantly, based on the edge betweenness centrality analysis, we believe that blood MMP-9 may play an important role in affecting the brain in-line with our earlier result (Figure 3A) and consistent with an earlier publication (37).

To determine which biochemical components are significant with regards to the overall network, we performed nodal analyses involving stress, betweenness, closeness, radiality, and eccentricity centralities. As shown in Figure 7B, only blood NADPH and blood CASP1 components are considered significant in all centralities (stress, betweenness, closeness, eccentricity, and radiality) studied which we believe would insinuate that variations in the levels of these two components may likewise affect the overall network. Coincidentally, this is consistent with CASP1 and NADPH function (28, 33). Additionally, we postulate that the following components are important (based on stress), crucial (based on betweenness), and relevant (based on both closeness and radiality) to the overall network: blood H_2_O_2_, blood MMP-9, blood TLR2, brain heme, brain NADPH, and brain H_2_O_2_. Noticeably, these components were not significant in terms of eccentricity centrality which would suggest that these components do not change easily, since these components play an important physiological role and sudden alterations could be detrimental and harmful (24, 28, 37, 38, 55). Interestingly, both brain GADD153 and brain calcium were only considered important (based on stress) and crucial (based on betweenness). We believe that both GADD153 and calcium have significant physiological roles which would emphasize the importance of maintaining homeostasis in order to avoid any pathological effects (46, 56). Additionally, based on eccentricity centrality, brain calcium is suggested to be easily influenced. We associated this to calcium being easily affected by both internal and external cellular factors (49, 57).

## Discussion

Periodontal disease level-butyric acid contributes to PD development, while, PD influences SD development which in turn could affect certain organs (2, 5, 7). Throughout this study, we attempted to establish the putative systemic manifestations in the blood and organ-localized effects in the brain associated with gingival PDL-BA. Moreover, we elucidated the possible sequential networks linking the effects of gingival PDL-BA to the brain organ.

Among the numerous bacterial and viral communities colonizing the periodontal pocket, Gram-negative bacteria are considered the most virulent communities involved in PD development (58). PD treatment has been postulated to alleviate certain SD (2, 59) highlighting the possible role of PD as a risk factor for several SD (12). Moreover, inflammation was found to play an important role in SD development, particularly involving the blood and brain (60). This would imply that PD-related inflammation could serve as a risk factor for SD development. Inflammation during the early PD stages has been attributed to physiologic defense, while inflammation found in the later PD stages has been ascribable to pathology (1). In this regard, we hypothesize that late stage PD-related inflammation is involved in SD development.

It was previously suggested that the host microbiota could have extensive regulatory effects on host physiology and function that may affect all organ systems (61). Among the various possible microbial components that could affect the host, SCFAs have been mainly attributed to several microbe-to-host signaling (62) further emphasizing the importance of bacterial metabolites, such as BA (63).

Detectable BA levels circulate and prolong in the blood (9, 10). However, in this study BA was no longer detectable in the blood. Nevertheless, NADPH-related oxidative stress and putative MMP-9-modulated inflammation were induced. In this regard, we postulate that gingival PDL-BA has prolonged systemic manifestations. Similarly, increased MMP-9 activation resulted in a simultaneous increase in TLR2 activity which in turn leads to increased protein amounts among the three representative neurological blood biomarkers studied (APP, PS1, and PS2). This implies that gingival PDL-BA could have detrimental neurological effects. Varying BA concentrations have previously been shown to affect different organ systems (64) which would insinuate that at PDL-BA levels, asides from contributing to PD development, other organ systems could likewise be affected. Among the different organ systems, the central nervous system has been most affected by bacterial presence, metabolism, and function (65). Consistently, our results showed that gingival PDL-BA can likewise affect the different brain regions *via* cellular (oxidative and ER) stress induction. Moreover, increase in cellular stress signaling resulted in increased calcium signaling and cell death activities in the different brain regions which would indicate that the brain organ is affected by gingival PDL-BA regardless of having no detectable BA concentration. This further highlights the prolonged organ-localized effects of PDL-BA. Similarly, this demonstrates that in order to affect brain function, BA need not be present in the brain (66). This would imply that gingival PDL-BA has an indirect effect to the brain which we hypothesize involves several components. Additionally, total tau protein amounts were increased only in the hippocampal and cerebellar brain regions which coincidentally make up the cerebellar–hippocampal interaction involved in motor control and cognitive function (67). In this regard, we presumed that increase in brain tau protein amounts ascribable to gingival PDL-BA may have a detrimental neurological effect. Considering the PDL-BA-related route affected (gingival–blood-brain), we postulate that during a PD scenario, wherein, late stage PD-related inflammation occurs (1). PDL-BA is potentially involved in systemic inflammation, since multiple biochemical components are involved and different phase-specific (gingival–blood–brain) pathologic processes are concurrently developing (68). In this regard, we propose that the possible PDL-BA-associated systemic inflammation would entail the sequential network involving blood NADPH, blood H_2_O_2_, blood MMP-9, brain heme, brain NADPH, brain H_2_O_2_, brain GADD153, and brain calcium. This in turn induced blood oxidative stress leading to blood MMP-9-modulated inflammation resulting to brain oxidative and ER stresses which, consequently, affected the different brain regions with both the cerebellar and hippocampal regions being most affected.

## Conclusion

In summary, we attempted to show the unfavorable systemic effects of BA, particularly gingival PDL-BA. In the blood, we hypothesize that NADPH-related oxidative stress lead to inflammation putatively modulated by MMP-9. We attributed this to the probable prolonged PDL-BA effects. Similarly, in the brain, we postulate that NADPH-related oxidative stress lead to ER stress which in turn potentially induced calcium and cell death signaling resulting to elevated tau protein amounts in the cerebellar and hippocampal regions. We suspect that this has a detrimental effect to the brain that could potentially result to neurological disorder. Taken together, we propose that gingival BA can potentially cause systemic inflammation ascribable to prolonged systemic manifestations in the blood and localized detrimental effects within the brain.

## Ethics Statement

This study was carried out in accordance with the recommendations of “Kyoto Institute of Nutrition and Pathology.” The protocol was approved by the “Kyoto Institute of Nutrition and Pathology.”

## Author Contributions

MC performed the experiments and co-wrote the paper. KO co-wrote the paper and aided in the analyses of results.

## Conflict of Interest Statement

The authors declare that the research was conducted in the absence of any commercial or financial relationships that could be construed as a potential conflict of interest.
